# Effect to Therapy of Sodium-Iodine Symporter Expression by Alpha-Ray Therapeutic Agent via Sodium/Iodine Symporter

**DOI:** 10.3390/ijms232415509

**Published:** 2022-12-07

**Authors:** Kazuko Kaneda-Nakashima, Yoshifumi Shirakami, Tadashi Watabe, Kazuhiro Ooe, Takashi Yoshimura, Atsushi Toyoshima, Yang Wang, Hiromitsu Haba, Koichi Fukase

**Affiliations:** 1 Core for Medicine and Science Collaborative Research and Education, Forefront Research Center, Osaka University Graduate School of Science, 1-1 Toyonaka, Osaka 560-0043, Japan; 2 Institute for Radiation Sciences, Osaka University, 2-4 Suita, Osaka 565-0871, Japan; 3 Department of Nuclear Medicine and Tracer Kinetics, Osaka University Graduate School of Medicine, 2-2 Suita, Osaka 565-0871, Japan; 4 Nishina Center for Accelerator-Based Science, RIKEN, 2-1 Hirosawa Wako, Saitama 351-0198, Japan; 5 Department of Chemistry, Graduate School of Science, Osaka University, 1-1 Toyonaka, Osaka 560-0043, Japan

**Keywords:** NIS expression, [^211^At]NaAt therapy, [^131^I]NaI therapy, thyroid cancer, alpha therapy

## Abstract

This study confirmed the effect of sodium/iodine symporter (NIS) expression on existing drugs by in vitro and in vivo tests using cultured cell lines. The tumor growth inhibitory effect of sodium astatide ([^211^At]NaAt) was evaluated by in vitro and in vivo tests using human thyroid cancer cells (K1, K1/NIS and K1/NIS-DOX). NIS expression in cancer cells was controlled using the Tet-On system. [^131^I]NaI was used as control existing drug. From the results of the in vitro studies, the mechanism of [^211^At]NaAt uptake into thyroid cancer cells is mediated by NIS, analogous to [^131^I]NaI, and the cellular uptake rate correlates with the expression level of NIS. [^211^At]NaAt’s ability to inhibit colony formation was more than 10 times that of [^131^I]NaI per becquerel (Bq), and [^211^At]NaAt’s DNA double-strand breaking (DSB) induction was more than ten times that of [^131^I]NaI per Bq, and [^211^At]NaAt was more than three times more cytotoxic than [^131^I]NaI (at 1000 kBq each). In vivo studies also showed that the tumor growth inhibitory effect of [^211^At]NaAt depended on NIS expression and was more than six times that of [^131^I]NaI per Bq.

## 1. Introduction

The large uptake of iodine by the thyroid gland has provided opportunities for the use of [^131^I]NaI in radioactive iodine therapy for patients with differentiated thyroid cancer [1,2,3,4]. However, a number of patients do not show sufficient therapeutic effects with [^131^I]NaI therapy, even with sufficient uptake of [^131^I]NaI [4,5]. For these patients, we considered an alpha-ray therapy targeting the sodium/iodide symporter (NIS) as a more effective therapy. Short-lived alpha rays have a short range and strong cytotoxicity, which makes them suitable for treatment. We are focused on astatine-211 (^211^At, half-time: 7.2 h), an alpha-emitting nuclide that can be produced in an accelerator.

We have already started clinical trials of [^211^At]NaAt to solve the problems associated with [^131^I]NaI in radioiodine therapy at Osaka University Hospital. However, although it has been reported that [^211^At]NaAt uptake is via NIS, there are no clear data on whether there is a correlation between the expression level of NIS and therapeutic effects. The purpose of this study was to evaluate the influence of NIS expression on the therapeutic effects of [^131^I]NaI and [^211^At]NaAt, even in a model with low expression of NIS.

One of the reasons for the failure of RAI treatment is the decreased expression of NIS. Various mechanisms have been proposed as the cause of NIS decrease [6], but in any case, it is speculated that when the expression of NIS decreases, the amount of nuclide uptake decreases and the therapeutic effect decreases. In the case of reduced NIS expression, it may be necessary to use nuclides with high biological efficacy [7]. Investigation of the correlation between NIS expression and therapeutic efficacy is also of great importance in confirmation the usefulness of astatine.

## 2. Results

### 2.1. Examination of Uptake, Colony-Forming Ability and DSB Induction of K1-NIS/DOX Cells Whose NIS Expression Level Was Controlled by Tet-On System

**Figure 1:**
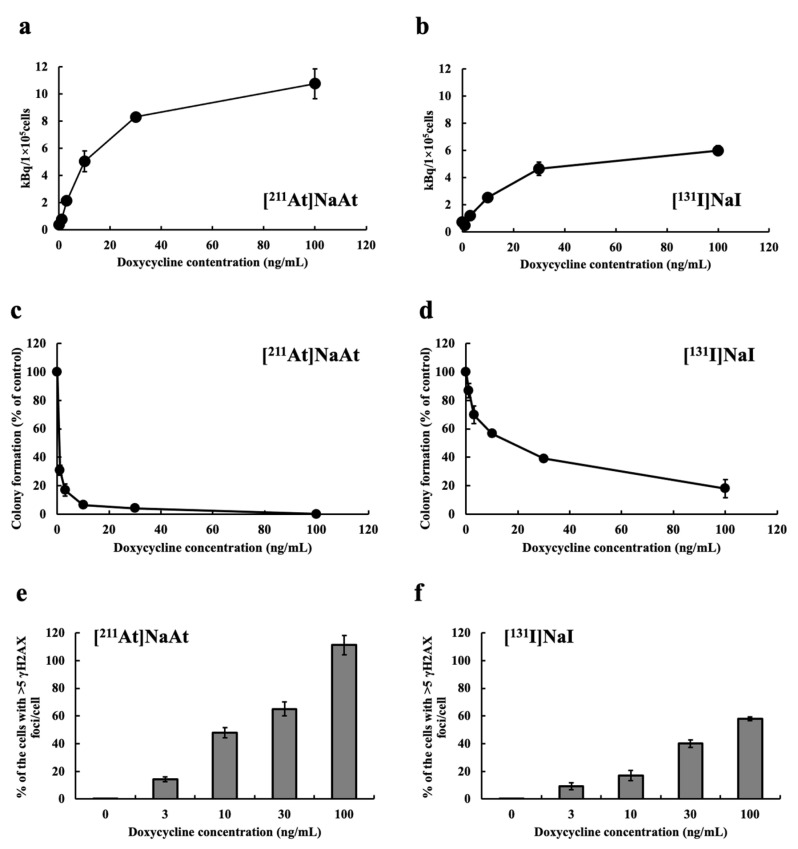
Uptake amount of K1-NIS/DOX cells treated with (**a**) [^211^At]NaAt and (**b**) [^131^I]NaI solutions. Colony formation (% of control) treated with (**c**) [^211^At]NaAt and (**d**) [^131^I]NaI solutions. % Of the cells with >5 γH2AX foci/cell treated with (**e**) [^211^At]NaAt and (**f**) [^131^I]NaI solutions. Data is shown in mean ± S.E.

It had already confirmed that the expression of NIS in K1-NIS/DOX cells increases according to the amount of doxycycline treated. The uptake of [^211^At]NaAt by K1-NIS/DOX cells was nearly double that of [^131^I]NaI (Figure 1a,b). For both nuclides, colony formation inhibition increased with increasing DOX concentration (i.e., increasing NIS expression level) (Figure 1c,d). [^211^At]NaAt was more than ten times more effective than [^131^I] NaI in inhibiting colony formation. The uptake of both nuclides is considered to be dependent on the expression levels of NIS. The frequency of DSB increased with increasing DOX concentration (NIS expression level) for both nuclides, and was compared using % of cells with >5 γH2AX foci/cell. Despite the [^211^At]NaAt treatment of 1/10, the radioactivity of [^131^I]NaI strongly caused damage to the cells (Figure 1e,f).

### 2.2. Comparison of Cytotoxically Effects in K1 Cells with and without NIS

**Figure 2:**
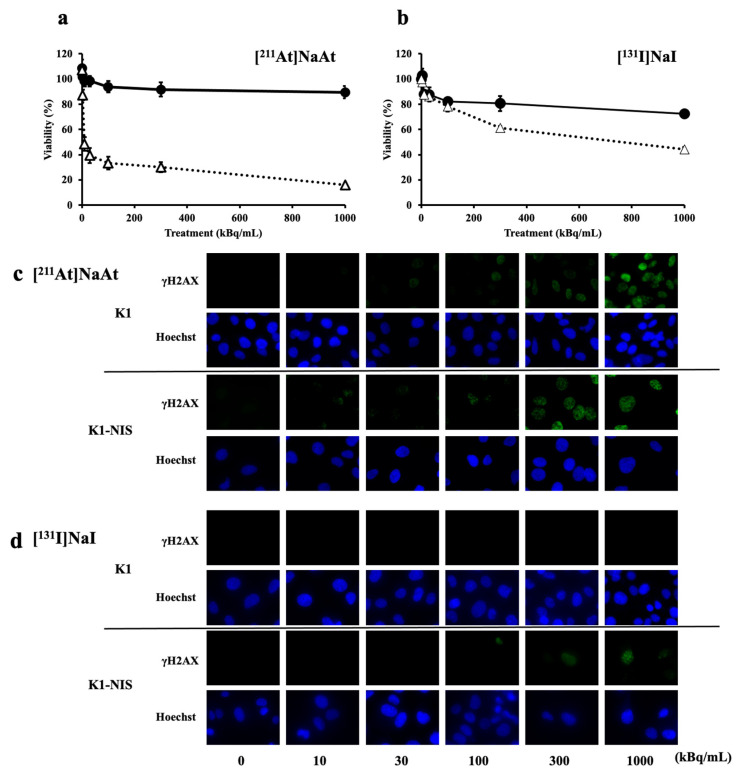
Viability of K1 or K1-NIS cells treated with [^211^I]NaAt (**a**) and [^131^I]NaI (**b**) solutions. Black circles represent K1 cells and white triangles represent K1-NIS cells. Data was shown in mean ± S.D. DSB induction of K1 or K1-NIS cells treated with [^211^I]NaAt (**c**) and [^131^I]NaI (**d**) solutions. Green fluorescence indicates γH2AX and blue fluorescence indicates nuclei.

Using K1 and K1-NIS cells, we confirmed the cytotoxically effects of NIS. NIS-independent cytotoxicity was lower for [^211^At]NaAt. In contrast, NIS-dependent cytotoxicity was higher for [^211^At]NaAt (Figure 2a,b). Adjusting the amount of radioactivity induces DSBs with [^211^At]NaAt at doses that do not induce DSBs with [^131^I]NaI. It was found that [^211^At]NaAt caused more potent cytotoxicity than [^131^I]NaI because [^211^At]NaAt imparts energy locally (Figure 2c,d).

### 2.3. Comparison of Cellular Uptake in K1 Cells with and without NIS

**Figure 3:**
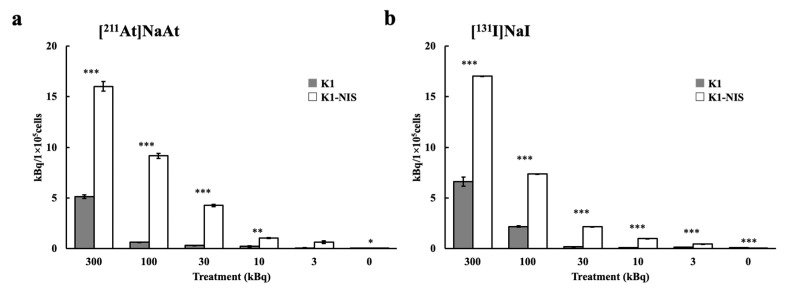
Uptake of K1 or K1-NIS cells treated with [^211^I]NaAt (**a**) and [^131^I]NaI (**b**) solutions. Data was shown in mean ± S.E. *p*-value: *** *p* < 0.001, ** *p* < 0.01. * *p* < 0.05.

In both the [^131^I]NaI and [^211^At]NaAt groups, there was a positive correlation between cell uptake and radioactivity added per well, indicating that both nuclides were almost identical. K1-NIS cells showed a radioactivity uptake more than three times that of K1 cells (Figure 3a,b). These results indicate that the cellular uptake of [^131^I]NaI and [^211^At]NaAt is due to a specific NIS-mediated mechanism and that the affinities of both nuclides for NIS are almost identical.

### 2.4. Colony-Forming Ability

**Figure 4:**
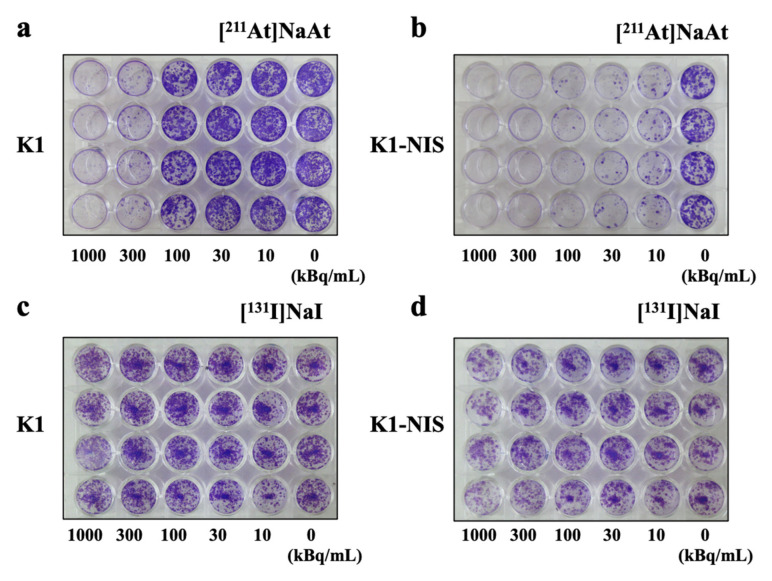
K1 or K1-NIS cell images after treatment with [^211^I]NaAt (**a**,**b**) and [^131^I]NaI (**c**,**d**) solutions stained with crystal violet.

In the case of [^131^I]NaI, almost no decrease in the colony-forming ability was observed in any well (Figure 4c,d). This difference is clear when compared with the decrease in the [^211^At]NaAt wells. [^211^At]NaAt-treated K1-NIS cells showed a marked decrease in colony-forming ability in all wells, except the control wells (0 kBq). [^211^At]NaAt-treated K1 cells inhibited colony formation only in wells with radioactivity ≥ 300 kBq (Figure 4a). These results showed that remarkably, [^211^At]NaAt inhibited colony-forming ability and was affected by the presence or absence of NIS expression. However, since the colony-forming ability of K1 cells is also suppressed by high radioactivity (300 kBq or more), a small amount of NIS may be expressed in K1 cells; that is, even with a small amount of NIS expression the non-specific effects of [^211^At]NaAt in the high radioactivity group cannot be completely denied (Figure 4b).

### 2.5. Contribution of NIS in Therapeutic Efficacy

**Figure 5:**
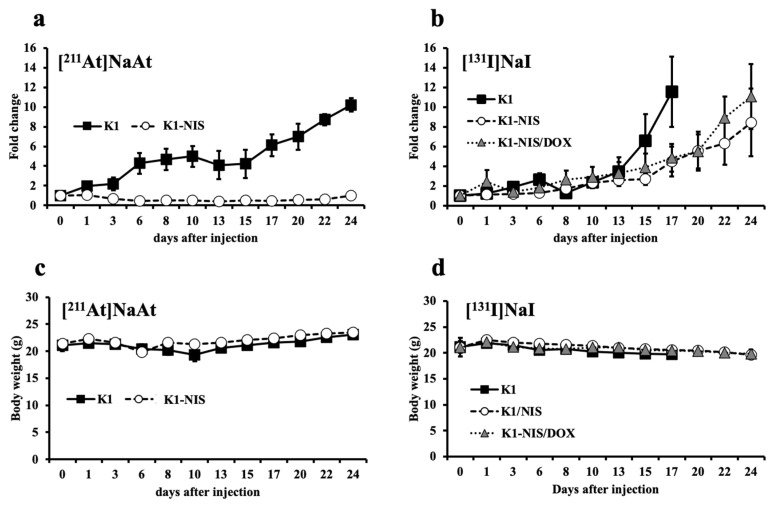
Follow up of tumor bearing model of K1, K1-NIS, and K1-NIS/DOX cell treated [^211^I]NaAt (**a**,**c**) and [^131^I]NaI (**b**,**d**) solutions. Tumor sizes were shown in (**a**,**b**), and weights were shown in (**c**,**d**). Data was shown in mean ± S.D.

The therapeutic effects of NIS on tumors with and without NIS were compared. Tumors with a higher expression of NIS (K1-NIS) were more efficacious. In addition, [^211^At]NaAt showed a stronger inhibitory effect on tumor growth (Figure 5a,c). Compared to [^131^I]NaI (Figure 5b,d), [^211^At]NaAt also inhibited the growth of K1 tumors.

## 3. Discussion

In vitro studies revealed that the uptake mechanism of [^211^At]NaAt in thyroid cancer cells, such as [^131^I]NaI, was mediated by NIS, and cytotoxicity was correlated with the expression level of NIS. The colony formation inhibition of [^211^At]NaAt was more than ten times that of [^131^I]NaI, and [^211^At]NaAt DSB was also more than ten times that of [^131^I]NaI; [^211^At]NaAt was more than three times more cytotoxic than [^131^I]NaI (1000 kBq). In vivo studies have shown that the tumor growth-inhibitory effect of [^211^At]NaAt depends on the amount of NIS expressed in human thyroid carcinomas and is more than six times (per Bq) that of [^131^I]NaI.

Previous reports suggest that the expression of NIS often decreases with differentiation, thus reducing the effects of radioactive iodine ([^131^I]NaI) therapy. In this experiment, it was confirmed that the expression of NIS contributed significantly to treatment with [^131^I]NaI or [^211^At]NaAt. However, while NIS expression was strongly important in the effect of RAI therapy, notable therapeutic effects were observed in [^211^At]NaAt therapy, even in cells with low NIS expression (K1). In other words, it was suggested that [^211^At]NaAt is effective even in patients with a low response to [^131^I]NaI. However, the transport mechanism of [^211^At]NaAt still remains unclear. Initially, it was thought that astatine might also be transported to tumors by symporters other than NIS, however, the results of uptake experiments indicated that the main reason for the high therapeutic effect of astatine was the high cellular effect of alpha rays.

To prevent non-specific accumulation of [^211^At]NaAt, elemental blockade is considered effective, as is treatment with iodine [8]. In addition, [^211^At]NaAt is highly accumulated not only in the thyroid but also in the stomach [9,10,11]. This is believed to be due to NIS expression. Of course, it is possible that the chlorine transporter for producing gastric juice is active, but this has not been verified. Since the authors previously confirmed that [^211^At]NaAt accumulation was suppressed using H2 blockers [data not shown], it is necessary to investigate the involvement of transporters other than NIS in [^211^At]NaAt transport.

Although NIS expression in K1-NIS/DOX was easily controlled with doxycycline in vitro, it was more difficult to control the expression levels of NIS in K1-NIS//DOX in vivo. This is because K1 and K1-NIS have distinctly different proliferation rates, and drug-treated cells consist of a heterogeneous population. However, it is possible that the heterogeneous population may mimic clinical patient tissue. Our model was preliminary; thus, we attempted to establish an animal model.

## 4. Materials and Methods

### 4.1. Preparation of [^211^At]NaAt and [^131^I]NaI Solution

^211^At was acquired from the National Institute for Quantum Science and Technology (QST) and RIKEN through a supply platform of short-lived radioisotopes. ^211^At was produced according to the ^209^Bi(α, 2n)^211^At reaction and separated from the Bi target using the dry distillation method. The separated ^211^At was then dissolved in pure water. Ascorbic acid (used as a reducing agent) and sodium bicarbonate (used as a pH adjuster) were added to the crude ^211^At solution to a final concentration of 1% (*w*/*v*) at pH 8.0, and the solution was allowed to stand for 1 h at 23 ± 2 °C. The concentration of At was 10 MBq/mL.

Solutions of [^131^I]NaI were purchased from the Japan Radioisotope Association (JRIA). Ascorbic acid (used as a reducing agent) and sodium bicarbonate (used as a pH adjuster) were added to the ^131^I solution to a final concentration of 1% (*w*/*v*) at pH 8.0, and the solution was allowed to stand for 1 h at 23 ± 2 °C. The concentration of I was 50 MBq/mL.

### 4.2. In Vitro Observation of DSBs of DNA and Colony Formation Assay

The human papillary thyroid carcinoma cell line K1 was purchased from the European Collection of Authenticated Cell Cultures. NIS expression was induced by transfecting K1 cells with the human SLC5A5 (NIS) gene clone (OriGene Technologies, Inc., Rockville, MD, USA). K1-NIS/DOX cells were established using a Tet-on 3G system (Takara Bio Inc., Kusatsu, Shiga, Japan). A tetracycline-dependent NIS expression cassette was retroviral and was used after cloning. The expression of NIS was controlled by treatment with doxycycline (DOX). K1-NIS cells were cultured in a mixed medium of DMEM (FUJI-FILM Wako Pure Chemical Corp., Osaka, Japan), Ham’s F12 (FUJIFILM Wako Pure Chemical), and MCDB 105 (Merck KGaA, Darmstadt, Germany) (2:1:1) supplemented with 10% heat-inactivated fetal bovine serum (Thermo Fisher Scientific, Inc., Waltham, MA, USA), 2 mM glutamine (FUJIFILM Wako Pure Chemical), and 1% penicillin–streptomycin solution (FUJIFILM Wako Pure Chemical). To maintain K1/NIS-DOX, we used Tet-System Approved FBS (Takara Bio Inc.).

To measure DSBs, K1-NIS cells were seeded in an eight-well chamber slide at a density of 1 × 10^5^ cells/mL. After two days of incubation, the cells were treated with 10 μL medium/well as the control group; 10, 30, 100, 300, and 1000 kBq [^211^At]NaAt solution/well as ^211^At groups; and 10, 30, 100, 300, and 1000 kBq [^131^I]NaI solution/well as ^131^I groups for 20 min. The volume of the solution was approximately 325 μL/well during treatment. After washing with phosphate-buffered saline (PBS), the cells were stained using the HCS DNA Damage Kit (Thermo Fisher Scientific, Inc., Waltham, MA, USA). The fluorescence signals were observed using a fluorescence microscope (BZX-810; Keyence Corporation, Osaka, Japan). The ability to induce DSBs was calculated as the percentage of cells with >5 γH2AX foci/cells treated with both solutions. Colony formation was calculated using ImageJ software and compared between the groups. Cells of interest were selected, and areas of nuclear morphology (Hoechst 33342) and DNA damage (pH2AX antibody) were observed.

K1, K1-NIS, and K1-NIS/DOX cells were seeded in 24-well plates to 70–80% confluence and detached for the colony formation assay. The cells in each well were treated with 0, 10, 30, 100, 300 and 1000 kBq [^211^At]NaAt solution and 0, 10, 30, 100, 300 and 1000 kBq [^131^I]NaI solution. The volume of the solution was 0.5 mL. After 1 h of treatment at 37 °C in a humidified atmosphere of 5% CO_2_, cells were counted and seeded in fresh medium in 24-well plates at a density of 500 cells/well. After 14 days of incubation, the cells were fixed and stained with a crystal violet solution. The cells were viewed and counted under a microscope (Primo Star, Carl Zeiss AG, Oberkochen, Germany).

### 4.3. Preparation of Animals

Male nude mice were purchased from Japan SLC, Inc. (Hamamatsu, Shizuoka, Japan), housed under a 12-h light/12-h dark cycle, and allowed free access to food and water. The mice were injected with K1, K1-NIS or K1-NIS/DOX cells (1 × 10^7^ cells) in 0.2 mL of culture medium and Matrigel (1:1; BD Biosciences, Franklin Lakes, NJ, USA) into the right flank. The tumor size was approximately 50 mm^3^, with a growth phase of 4 weeks, before the administration of [^131^I] NaI or [^211^At] NaAt solution.

### 4.4. In Vitro and In Vivo NIS Control Model

For For NIS control experiments, K1-NIS/DOX cells were cultured in special serum to eliminate the effects of tetracycline analogs in normal serum. One day before nuclide treatment, the K1-NIS/DOX cells were treated with doxycycline at the determined concentrations. In the K1-NIS/DOX tumor-bearing model, doxycycline was intraperitoneal administrated two days before the injection (2 mg/mouse). Doxycycline was administered every two days, while [^131^I]NaI was expected to remain in the body.

### 4.5. Therapy with [^131^I]NaI and [^211^At]NaAt Solutions

Mice injected with [^131^I]NaI solution into the tail vein were divided into three groups, according to the tumor type: 5 MBq ^131^I group (5.00 ± 0.19 MBq, N = 3, 3 groups), K1 group (21.17 ± 1.14 g), K1-NIS group (21.10 ± 0.96 g), and K1-NIS/DOX group (21.07 ± 1.80 g). Mice injected with [^211^At]NaAt solution into the tail vein were divided into the following two groups, according to the tumor type: 0.8 MBq ^211^At group (0.80 ± 0.01 MBq, N = 3, 2 groups), K1 group (21.07 ± 1.01 g), K1-NIS group (21.37 ± 0.43 g). The tumor size and body weight were measured.

### 4.6. Statistical Analysis

Results are expressed as the mean ± standard deviation. Comparisons between groups were performed using unpaired *t*-tests in Microsoft Excel (version 2016, Microsoft Corp., Redmond, WA, USA). For multiple comparisons among the three groups, Bonferroni correction was performed. Differences were considered statistically significant at *p* < 0.05.

## 5. Conclusions

In this study, ^211^At showed effective DSBs induction with higher cellular toxicity, and the administration of [^211^At]NaAt was more effective in an NIS-expressing thyroid cancer model than the administration of [^131^I]NaI. These results suggest that [^211^At]NaAt therapy is a more promising option than [^131^I]NaI treatment for patients with iodine-avid thyroid cancer refractory. It was also confirmed that the amount of uptake was proportional to the expression level of NIS and that the therapeutic effect was also proportional to the expression level of NIS. However, with [^211^At]NaAt, a certain degree of therapeutic effect was observed even when the expression level of NIS was low and no side effects were observed, indicating the usefulness of [^211^At]NaAt.

## Data Availability

All data are included in this published article.
